# Blueprinting the ecosystem health index for blue carbon ecotones

**DOI:** 10.1016/j.isci.2024.111426

**Published:** 2024-11-26

**Authors:** Jiaqi Zhang, Matteo Convertino

**Affiliations:** 1 Center for Ecosystem Design and fuTuRE EcoSystems Lab (TREES), Institute of Environment and Ecology, Tsinghua Shenzhen International Graduate School, Tsinghua University, Shenzhen, China; 2 Shenzhen Key Laboratory of Ecological Remediation and Carbon Sequestration, Tsinghua SIGS, Shenzhen, China

**Keywords:** Environmental science, Ecology, Biogeoscience, Global carbon cycle

## Abstract

Blue carbon ecotones (BCEs) play a critical role in regulating abiotic and biotic ecological fluxes underpinning services which are also crucial for the protection of the land-ocean function.

Here, we proposed a Benefit-Pressure-Transformation Risk model (BPT) to calculate the Ecosystem Health Index (EHI) for mangrove, salt marsh, and seagrass as core BCEs globally (at a resolution of 1° × 1 °lat-long), based on habitat structure, species morphological features and vulnerability, niche overlap, nature and human pressures, and ecosystem services. Our assessments identify that around 20% of BCEs as vulnerable globally. Specifically, with every 1° increase in latitude, the EHI values for mangroves, salt marshes, and seagrasses decrease linearly proportionally to a factor equal to 0.007, 0.003, and 0.004.

We find that improving EHI of mangroves not only supports resilience but also enhances their carbon sink function (+68 Mg per hectare/0.1 EHI), making ecosystem health a critical factor in climate change mitigation.

## Highlights

• A Benefit-Pressure-Transformation Risk model calculates the Ecosystem Health Index
• ∼20% of BCEs are vulnerable globally, emphasizing the need for targeted conservation efforts
• Improving EHI of mangroves enhances their carbon sink function (+68 Mg/hectare/0.1 EHI)
• EHI of BCEs is more sensitive to structural indicators compared to environmental pressure

## Graphical abstract

**Figure:**
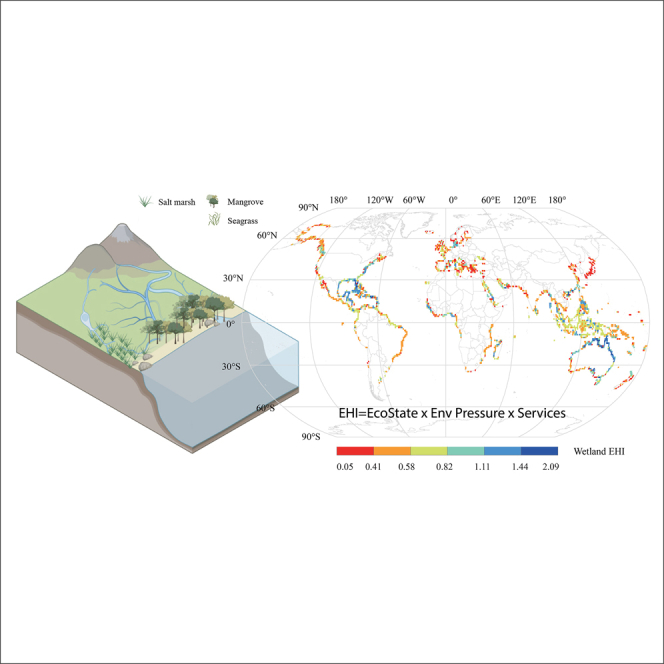

## Introduction

The concept of blue carbon was first introduced by Nellemann et al.,^1^ who described it as the carbon stored in autotrophic ecosystems such as mangroves, salt marshes, and seagrass beds that thrive on inorganic materials. Blue carbon ecotones are transitional areas where different blue carbon ecosystems (BCEs), such as mangroves, salt marshes, and seagrasses, meet. These zones play a vital role in connecting terrestrial and marine environments, facilitating nutrient exchange, and supporting the health of adjacent ecosystems. They cover <0.1% surface area of the earth, but are critical for climate change mitigation and ecosystem resilience through various mechanisms, including carbon sequestration, fish and marine species regulation, wave attenuation, and water purification.^2^^,^^3^^,^^4^^,^^5^ Sustainable Development Goal (SDG)-14 “Life Below Water” also highlights the potential of coastal wetlands to reduce carbon emissions and their roles in ensuring life on Earth is resilient when facing global climate change.^6^ Each of the core BCEs (mangroves, salt marshes, and seagrasses) has distinct biological and ecological characteristics that influence its distribution and response to environmental changes.^7^ For instance, mangroves prefer warmer temperatures and lower salinity environments, primarily thriving in tropical and subtropical estuarine regions.^8^ However, with global warming, studies suggest that mangroves are expanding into higher latitudes as freezing events become less frequent, leading to shifts in ecosystem boundaries.^9^ In contrast, salt marshes, although capable of surviving in higher salinity environments, are more sensitive to sea-level rise and tidal inundation, which affects their survival and distribution.^10^ Salt marshes are better adapted to temperate regions and exhibit a broader latitudinal range compared to mangroves. Finally, seagrasses, which are submerged plants, rely heavily on light availability for growth. While they can tolerate a wide range of temperatures, their low tolerance for freshwater environments limits their distribution in regions with reduced salinity.^11^

BCEs are under pressure from climate change and human activities,^12^^,^^13^ which affect their structure and function.^14^^,^^15^^,^^16^^,^^17^ Chowdhury et al.^18^ found that the presence of contaminants in mangrove ecosystems can disrupt ecological balance and reduce the overall health of the ecosystem. Dashtbozorgi et al.^19^ indicated that stresses caused by land use patterns have led to a general decline in habitat suitability for mangroves. An et al.^20^ indicated that sea level rise caused by global warming has led to the submergence and reduction in area coverage of BCEs in tundra regions. Although modeling results showed that coastal wetlands were not so fragile to sea level rise depending on their nature-based adaptation,^21^ their expansion and migration caused by sea level rise were limited by human activities.^22^ Therefore, creating a practical model for conducting global-scale health assessments of BCEs, and addressing these key conservation issues, is crucial for their effective conservation and sustainable utilization. It also can serve as a vital tool for informed decision-making, policy formulation, and targeted conservation actions for the benefit of both nature and human well-being.

Robert Costanza defined ecosystem health as *“the ability of an ecosystem to maintain its structure and function, as well as its capacity to recover from disturbance and adapt to change”*.^23^ In other words, it refers to the overall health condition and resilience of an ecosystem. When an ecosystem is in a healthy status, it can effectively carry out its essential functions and provide numerous benefits to both the environment and society. In published research literature, researchers have predominantly focused on single indicators to assess the status of BCEs. Indicators were used to describe BCEs, such as area, soil carbon content, and carbon emission.^24^^,^^25^^,^^26^ For example, seagrass health assessments focused more on species-level risks, highlighted by the International Union for Conservation of Nature (IUCN)’s 2014 Seagrass Red List. Furthermore, assessment methods based on fish populations^27^ and vegetation populations^28^^,^^29^ were widely used to evaluate the status of BCEs. Although previous methods could provide valuable information about the health status of BCEs, they may not capture the full complexity of ecotone dynamics and processes. In addition, BCEs have limited their assessments to regional scales rather than global ones. For example, salt marshes lack a dedicated global assessment, with most studies being regional, such as those from the European Environment Agency^30^ and U.S. National Wetlands Condition Assessment.^31^ Besides, Halpern et al.^32^ created a comprehensive method to quantify the health of the ocean and developed the Ocean Health Index (OHI), which consisted of 10 aspects of public goals. For example, the biodiversity goal assesses the variety of marine species, reflecting the ecosystem’s resilience and overall health. However, this assessment focuses solely on single Exclusive Economic Zones (EEZs) Single exclusive economic zones (EEZs) refer to specific maritime areas established by countries, extending up to 200 nautical miles from their coastlines. Within these zones, a nation has exclusive rights to explore and exploit marine resources, including fishing, oil, and gas among the countries. It does not fully account for the ecological significance of blue carbon ecotones, which represents the interface between land and ocean. Therefore, a comprehensive index that combines widely disparate metrics is needed to assess the health status of BCEs globally.

**Figure 1:**
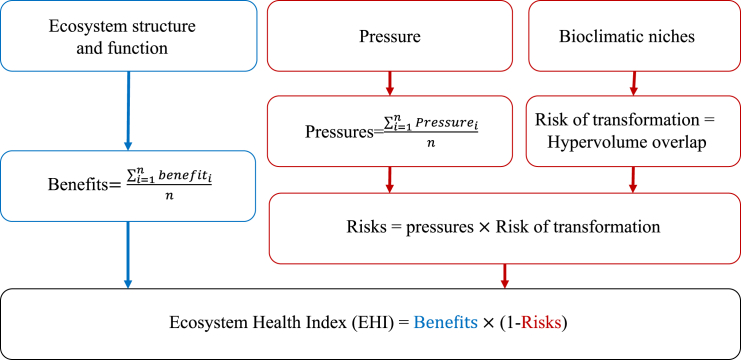
Framework for calculating Ecosystem Health Index

Mangroves have the most comprehensive evaluation compared to salt marshes and seagrasses. The IUCN’s 2024 global risk assessment divided the world’s mangroves into 36 ecosystems,^33^ and classified the ecosystems into seven risk categories qualitatively. Our proposed model aligns with this by not only assessing the risk of ecosystem collapse but also focusing on the resilience and functionality of BCEs. Based on existing coastal variables at pixel scale (1°(latitude(lat)) × 1°(longitude(long)) globally,^34^ we developed a Benefit-Pressure-Transformation Risk model (BPT model) to assess the health status of BCEs (mangroves, salt marshes, and seagrasses), which is defined as the Ecosystem Health Index (EHI) (Figure 1). The model consists of two parts: one part is to assess the benefit of BCEs (blue part of the framework) and the other is to assess the risk of BCEs (red part of the framework). This health assessment framework enables managers to monitor ecosystem services capacity and risk buffering functions of BCEs, especially in the context of global climate change.

Hutchinson introduced the concept of the multidimensional hypervolume in 1957 to quantify species’ ecological niche space.^35^ This concept has since been applied in the quantification of ecological niches. According to Darwin’s “struggle for existence”, the competitive exclusion principle states that “any two species occupying the same niche will compete with each other to the detriment of one of the species, which will thus be excluded.”^36^ Therefore, we held the view that high levels of ecological niche overlap (hypervolume overlap) indicated a greater likelihood of competition and potential displacement between BCEs (through habitat-exclusive invasion), which could lead to a loss of biodiversity, functional integrity, and overall ecotone health. We incorporated the concept of hypervolume overlap as a measure of the risk of transformation into our BPT model, which would provide a more comprehensive understanding of the health state of BCEs. In summary, assessing and visualizing the health of BCEs provides a scientific basis for ecological protection and policy-making, which enables proactive and evidence-based approaches to safeguarding the health and sustainability of BCEs.

This research aims to develop a comprehensive ecosystem health index that integrates multiple indicators of ecotone fitness, environmental pressures, and risk of transformation to provide a robust assessment framework for mangroves, salt marshes, and seagrasses. By addressing the limitations of existing studies, this work contributes valuable insights into the health and resilience of these critical ecosystems. This study has the potential to enhance our understanding of BCEs worldwide and guide future restoration initiatives, thereby contributing to climate change mitigation and the preservation of vital ecosystem services. The added value of this research lies in its ability to identify regional patterns of hypervolume overlap, which can inform targeted conservation efforts and management strategies tailored to specific geographical contexts.

## Results and discussion

### Mapping EHI for mangrove, salt marsh, and seagrass worldwide

**Figure 2:**
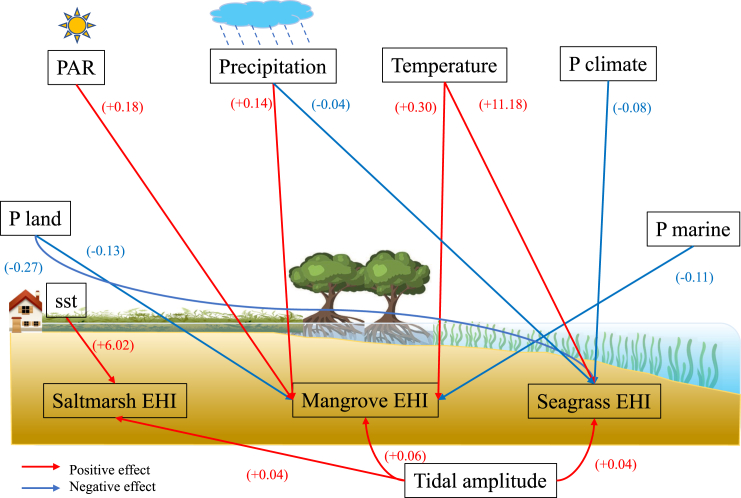
Conceptual graph of how environmental factors affect blue carbon ecotone EHI Red and blue arrows represent positive and negative impacts, and numbers represent mixed effects model regression coefficients for ecotones. PAR: Photosynthetic Active Radiation; Pland: Land Pressure; Pclimate: Climate Pressure.

**Figure 3:**
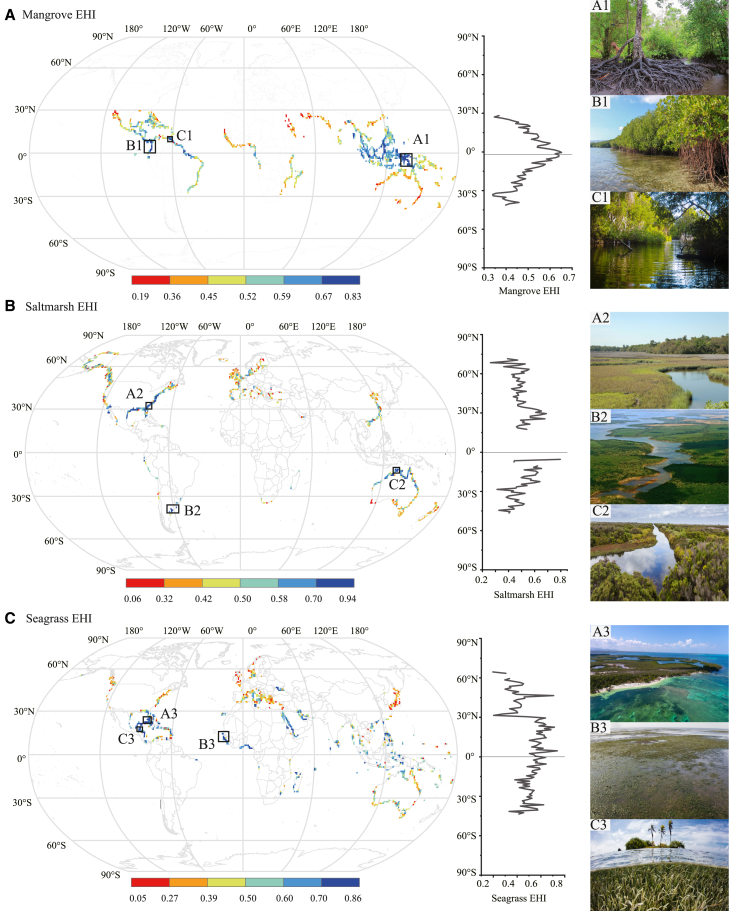
Global map and latitudinal pattern of blue carbon ecotone EHI (A) Global mangrove EHI map with the top three healthy hotspots highlighted. A1: Mangrove in Indonesia (Asia); B1: Mangrove in Colombia (South America); C1: Mangrove in Venezuela (South America). (B) Global salt marsh EHI map with the top three healthy hotspots highlighted. A2: Salt marsh on the East Coast of the U.S. (North America); B2: Salt marsh in Argentina (South America); C2: Salt marsh in northern Australia (Oceania). (C) Global seagrass EHI map with the top three healthy hotspots highlighted. A3: Seagrass in Cuba (North America); B3: Seagrass in Belize (North America); C3: Seagrass in Guinea-Bissau (West Coast of Africa). All photos were from Google.

**Figure 4:**
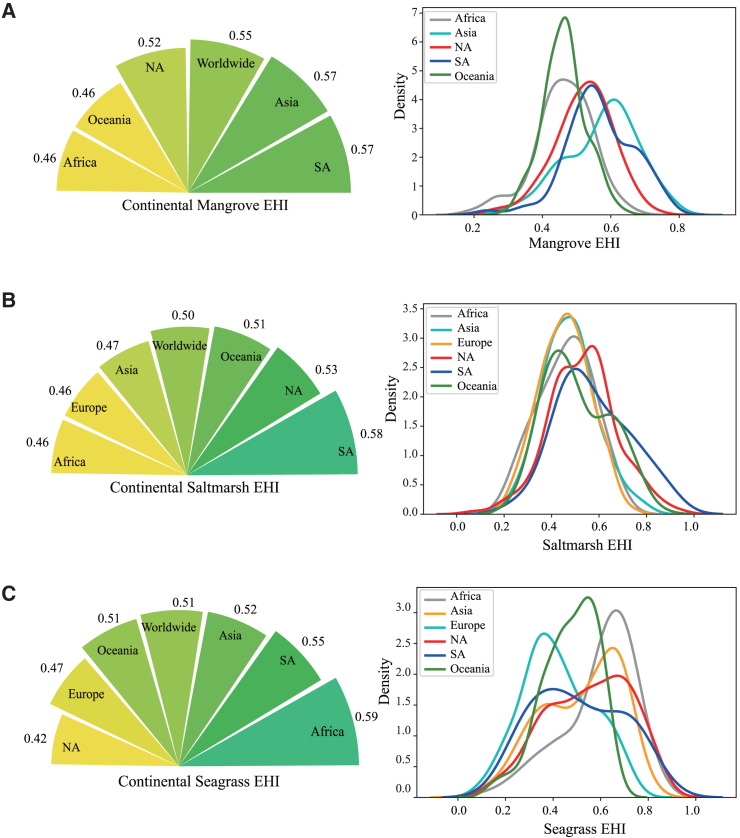
Mean value and probability distribution function (pdf) of blue carbon ecotone EHI for different continents (A) Mangrove; (B) Salt Marsh; (C) Seagrass.

Global mapping and latitude trend of EHI for BCEs, visualized in Figure 2 with their environmental determinants, are shown in Figure 3. Globally, mangroves are distributed mainly in tropical areas, between 38.5°S and 29.5°N (Figure 3A). To predict the global pattern of mangrove EHI values, we calculated the mean value of mangrove EHI along latitude, which showed that mangrove EHI decreased along latitude from the equator to the poles. As latitude increased by 1°, mangrove EHI decreased by 0.007 as a linear relationship factor (slope = −0.007, R square = 0.83, *p* value < 0.001, Figure S1A). Mangrove EHI calculated by our BPT model ranged from 0.19 to 0.83 (mean = 0.55) worldwide, and 22%, 43%, and 16% of mangroves are in high (>0.60), medium (0.45–0.60) and low (<0.45) health states, respectively. Additionally, 16% of the mangrove data were missing due to incomplete datasets from various sources (Table 1). The proportions of mangroves in high, medium, and low health states across different continents are shown in Figure S2A. The environmental pressure variables and niche overlap of BCEs are shown in Tables 2 and 3, respectively. The 16% of mangroves in the low EHI category closely aligns with the IUCN’s global risk assessment, which found that 19.6% of mangroves are at high risk, classified as either Endangered or Critically Endangered (CR), highlighting areas at severe risk of collapse.^33^ This close alignment between our model’s results and the IUCN’s assessment reinforces the robustness of our approach. The top three high mangrove EHI pixels are distributed in Indonesia (Asia), Colombia (South America) and Venezuela (South America) (Figure 3A). Moreover, mangrove EHI varies widely across continents, with South America (mean = 0.57 ± 0.0081), Asia (mean = 0.57 ± 0.0048), and North America (mean = 0.52 ± 0.0062) scoring highly followed by Africa (mean = 0.46 ± 0.0071) and Oceania (mean = 0.46 ± 0.0041) scoring poorly (Figure 4A; Table 4).

**Table 1:** Defined three classes for EHI by the natural breakpoint method with proportions

Ecotones	High	Medium	Low	no data
Mangrove EHI	<0.45	0.45–0.60	>0.60	–
	22%	43%	16%	16%
Salt marsh EHI	<0.42	0.42–0.59	>0.59	–
	21%	39%	24%	15%
Seagrass EHI	<0.39	0.39–0.61	>0.61	–
	22%	29%	20%	29%

**Table 2:** Compounding environmental pressure variables

Pressure Variable	Description
Climate Pressure	the sum of Ocean acidification (Oa), Sea surface temperature (Sst), and Sea level rise (Slr) for each coastal wetland site.
Land Pressure	the sum of Organic chemical pollution (Ocp), Direct human (Dp), and Nutrient pollution (Np) rasters for each coastal wetland site.
Marine Pressure	the sum of Artisanal fishing (Af), Commercial fishing Pelagic bycatch (low = CfPLb, high = CfPHb), Commercial fishing Demersal non-destructive bycatch (low = CfDnLb, high = CfDnHb), Commercial fishing Demersal destructive (Dd), Light (Ligt) and Shipping (Ship) rasters for each coastal wetland site.
Species Pressure	Proportion of IUCN threatened species within each coastal wetland site.

**Table 3:** Hypervolume overlap of BCEs globally and continentally

Hypervolume overlap	Worldwide	Africa	Asia	Europe	NA	SA	Oceania
Mangrove-Salt marsh	0.03	0.27	0.03	0	0.02	0.11	0.37
Mangrove-Seagrass	0.07	0.24	0.03	0	0.04	0.03	0.60
Salt marsh-Seagrass	0.50	0.36	0.49	0.48	0.47	0	0.58

The table shows all possible pairwise overlaps between ecotone pairs (2 × shared volume/summed volume).

**Table 4:** Mean ± SD values for Mangrove EHI, Saltmarsh EHI, and Seagrass EHI across continents

Continents	Mangrove	n	Saltmarsh	n	Seagrass	n
Africa	0.46 ± 0.0071	144	0.46 ± 0.0301	15	0.59 ± 0.0140	121
Asia	0.57 ± 0.0048	505	0.47 ± 0.0122	87	0.52 ± 0.0099	289
North America	0.52 ± 0.0061	201	0.53 ± 0.0082	308	0.42 ± 0.0109	195
South America	0.57 ± 0.0081	145	0.58 ± 0.0303	25	0.55 ± 0.0125	25
Oceania	0.46 ± 0.0041	231	0.51 ± 0.0108	168	0.51 ± 0.0362	178
Europe	no data	0	0.46 ± 0.0077	192	0.47 ± 0.0086	183

EHI: Ecosystem Health Index; SD: Standard Deviation.

Salt marshes are distributed in relatively high latitudes compared with mangroves globally and found in 3.5°S - 46.5°S and 17.5°*N* - 71.5°N (Figure 3B). The mean value of salt marsh EHI tends to decrease with increasing latitude. As latitude increased by 1°, salt marsh EHI decreased by 0.003 (slope = −0.003, R square = 0.26, *p*-value<0.001, Figure S1B). Salt marsh EHI calculated by our BPT model ranged from 0.06 to 0.94 (mean = 0.51) worldwide, and 21%, 39%, and 24% of salt marshes are in high (>0.59), medium (0.42–0.59) and low (textless 0.42) health states, respectively. Additionally, 15% of the salt marsh data were missing due to incomplete datasets from various sources (Table 1). The proportions of salt marshes in high, medium, and low health states across different continents are shown in Figure S2B. The top three high salt marsh EHI pixels are distributed on the east coast of the U.S. (North America), Argentina (South America), and northern Australia (Oceania) (Figure 3B). Moreover, salt marsh EHI exhibits significant variation across continents, with South America (mean = 0.58 ± 0.0303) having the highest scores, followed by North America (mean = 0.53 ± 0.0082), Oceania (mean = 0.51 ± 0.0108), Asia (mean = 0.47 ± 0.0122), Africa (mean = 0.46 ± 0.0301), and Europe (mean = 0.46 ± 0.0077) (Figure 4B; Table 4).

Among the three kinds of BCEs, seagrasses occupy the largest geographical range in terms of latitude, which was found between 43.5°S and 69.5°N (Figure 3C). The mean value of seagrass EHI tended to decrease with increasing latitude. As latitude increased by 1°, seagrass EHI decreased by 0.005 (slope = −0.005, R square = 0.49, *p* value < 0.001, Figure S1C). Seagrass EHI calculated by our BPT model ranged from 0.05 to 0.86 (mean = 0.50) worldwide, and 22%, 29%, and 20% of seagrasses are in high (>0.61), medium (0.39–0.61) and low (<0.39) health states respectively. Additionally, 29% of the seagrass data were missing due to incomplete datasets from various sources (Table 1). The proportions of seagrasses in high, medium, and low health states across different continents are shown in Figure S2C. The top three high seagrass EHI pixels are distributed in Cuba (North America), Belize (North America) and Guinea-Bissau (West coast of Africa) (Figure 3C). Moreover, seagrass EHI varies widely across continents, with Africa (mean = 0.59 ± 0.0140) scoring highly, followed by South America (mean = 0.55 ± 0.0125), Asia (mean = 0.52 ± 0.0099), Oceania (mean = 0.51 ± 0.0362), Europe (mean = 0.46 ± 0.0086), and North America (mean = 0.42 ± 0.0109) (Figure 4C; Table 4).

After assessing the health state of the three BCEs, global coastal wetland EHI was defined as the sum of Mangrove EHI, Salt marsh EHI, and Seagrass EHI (Figure S3). From the equator to higher latitudes, there was a decreasing trend in wetland EHI. In all, tropical areas had healthier coastal wetland ecotones than high-latitude areas.

### Global and continental hypervolume overlaps of Blue Carbon Ecosystems

Although we did not consider the spatial dependence among pixels when calculating EHI, understanding the overlap of hypervolume between BCEs is important as it provides insights into the potential interactions, connectivity, and ecological relationships. In this research, the results of the global hypervolume of mangrove, salt marsh, and seagrass showed that mangroves prefer environments with higher photosynthetic active radiation (PAR), sea surface temperature (SST), and air temperature, and occupying the smallest climatic hypervolume (3.83E-05) compared with seagrass (9.68E-04) and salt marsh (2.02E-03) (Figure 6). Globally, three kinds of BCEs had different hypervolume overlap with each other, 50% between seagrass and salt marsh, 7% between seagrass and mangrove, and 3% between mangrove and salt marsh. Due to variations in climatic conditions, there were significant differences in the hypervolume overlap of BCEs across different continents. In the calculation of EHI for global BCEs, we analyzed hypervolume overlap for BCEs continentally. This approach allowed us to obtain more accurate EHI results worldwide by considering the specific risk of transformation among BCEs within each continent, and the results of continental hypervolume overlap among BCEs are shown in Figures S4–S9 and Table 3.

**Figure 5:**
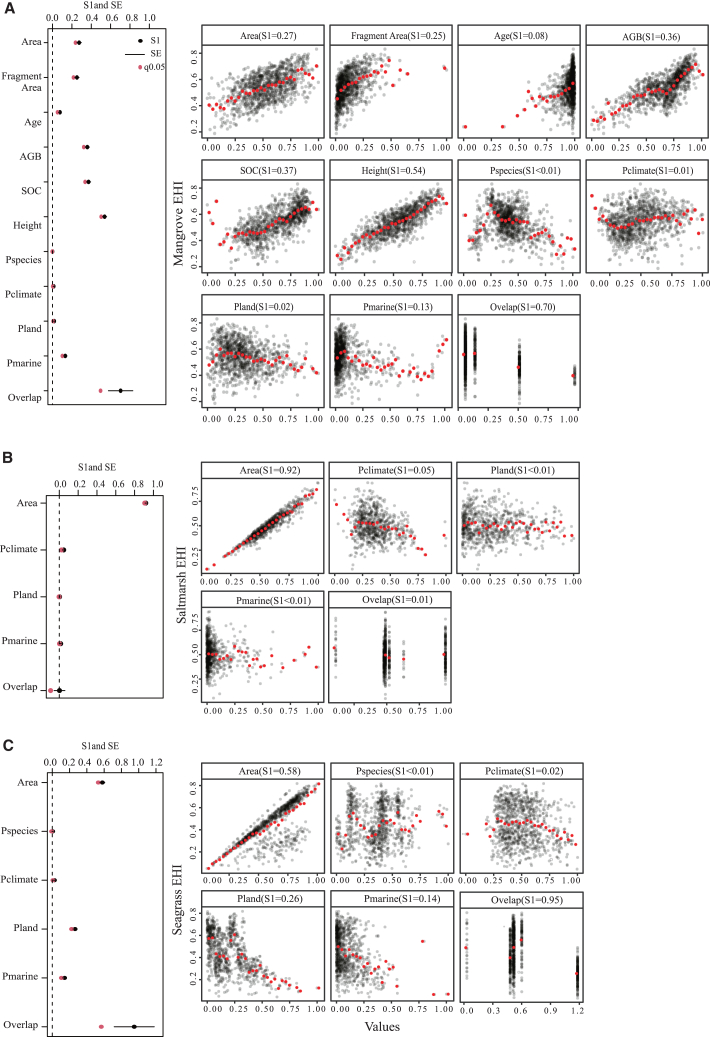
Estimated first order Sobol’ indices with standard deviations the calculated standard errors and scatterplot of EHI against parameters The estimated first-order Sobol’ indices; SE: the standard errors for the estimated first-order Sobol’ indices; q0.05: the 0.05 quantiles assuming Normal distributions for the S1 indices. The red dots show the mean EHI value in each bin (we have set the number of bins arbitrarily at 30); (A) Mangrove; (B) Salt Marsh; (C) Seagrass. AGB: Aboveground Biomass; SOC: Soil Organic Carbon; Pspecies: Species Pressure; Pland: Land Pressure; Pclimate: Climate Pressure; Pmarine: Marine Pressure.

**Figure 6:**
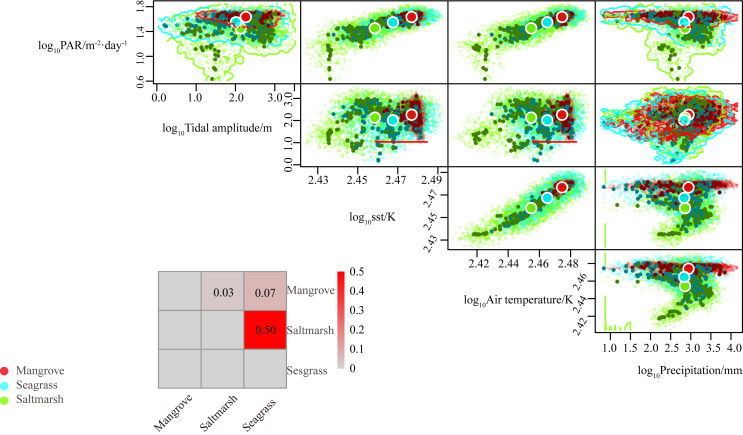
Pair plots for estimated climate hypervolumes of three blue carbon ecotones globally Red: Mangrove; blue: Seagrass; green: Salt Marsh. Hypervolumes were constructed using a Gaussian kernel density estimate with the Silverman method. All variables have been log10-transformed. The colored points for each ecosystem reflect the stochastic description of each hypervolume. The heatmap shows all possible pairwise overlaps between ecosystem pairs (2 × shared volume/summed volume).

The high hypervolume overlap (50%) globally between salt marshes and seagrass indicates bioclimatic similarity or ecotone overlap considering their spatial distribution. Researchers found that the mutual interaction between salt marsh and seagrass was complex. While they compete for resources, salt marshes and seagrasses also benefit from each other’s presence. For example, the presence of seagrass can reduce the flow of energy along the coastline, thereby increasing sediment deposition in intertidal areas, which benefits the survival of salt marshes.^37^ By filtering runoff and excess nutrients, salt marshes help maintain water quality in coastal areas, benefiting adjacent seagrass meadows.^38^ Both ecosystems are sensitive to inundation. As sea levels rise, areas that were once occupied by salt marshes may experience deep inundation, posing challenges to their survival.^10^ Simultaneously, this deep inundation can stress seagrasses due to limited light availability,^39^ potentially encouraging their landward expansion. The high hypervolume overlap indicates a greater risk of transformation under disturbance; however, the specific direction of this transformation is influenced by multiple factors such as sedimentation, eutrophication, and inundation.

Our results reveal significant variations in hypervolume overlap across different continents, highlighting how transformation risks and interactions between BCEs are regionally specific (Table 3). For instance, we find that in Oceania and Africa, the hypervolume overlap between mangroves and seagrasses reached 60% and 24%, respectively. This result is consistent with findings that indicate spatial overlap between mangroves and seagrasses in some tropical areas.^40^ We also find that Oceania shows a relatively high overlap between mangroves and salt marshes (0.37), likely due to both ecosystems occupying similar intertidal zones in coastal areas with mild climate transitions, allowing for some level of coexistence. This higher overlap indicates a greater risk of ecological transitions, where one BCE may shift into another, such as mangroves transforming into salt marshes under certain environmental changes, or vice versa. These regions may thus require targeted conservation strategies to manage these transitions and maintain ecosystem integrity. In contrast, Europe and North America exhibit lower overlaps between BCEs, primarily due to distinct climatic zones that limit the distribution of particular ecosystems. For instance, mangroves are largely confined to tropical regions, whereas salt marshes and seagrasses extend into temperate zones. This lower overlap suggests that ecosystems in these regions are more distinct, with fewer risks of transformation, and may have more stable boundaries. Therefore, conservation actions in these areas might focus more on protecting individual ecosystems from degradation rather than managing transitions between them. By identifying these regional patterns of hypervolume overlap, our study provides valuable insights for prioritizing conservation efforts tailored to specific geographical contexts.

### Exploring the determinants of EHI in blue carbon ecotones

To forecast the EHI of global blue carbon ecotones under diverse climatic factors, we employed a mixed-effects model to discern the impact of climatic factors and pressures from both nature and humans on EHI in our study. We identified that different BCEs exhibited varying sensitivities to different environmental conditions (Figure 2). These sensitivities stemmed from the unique characteristics and functional traits of each ecosystem type. For example, seagrasses exhibited a remarkable ability to dissipate wave energy, while salt marshes and mangroves excelled in providing protection against surges.^7^ The results of the mixed effects model in our results indicated that mangroves, with their ability to thrive in warmer climates with abundant rainfall,^8^^,^^41^ exhibited higher mangrove EHI to increasing PAR, temperature, and precipitation, and mangroves were primarily affected by land and marine pressures due to their proximity to coastal areas (Figure 2). Land-based pressures such as urbanization could result in increased sedimentation, and nutrient pollution in coastal waters, which negatively impacted mangrove ecotone.^42^ Additionally, coastal development and infrastructure could lead to habitat loss and fragmentation of mangroves. Marine pressures such as overfishing and destructive fishing practices also contributed to the degradation of mangrove habitats. The strong resilience of mangrove ecotone to climate pressure was supported by evidence showing that soil accretion rates within mangrove ecotone were currently keeping up with the rate of mean sea-level rise.^43^ This indicated that mangroves were effectively adapting to changing climatic conditions, demonstrating their ability to withstand the potential impacts of rising sea levels. Meanwhile, Wang et al.^44^ highlighted the significant role of mangroves in carbon sequestration, as temperature and precipitation rise, mangroves could exhibit an enhanced capacity to sequester carbon. The positive relationship between mangrove EHI and climatic indicators suggests that as global warming and precipitation increase, mangroves show improved health status and higher carbon accumulation. Additionally, these presented opportunities for mangroves to expand into temperate regions, as they benefited from the favorable conditions of increased temperature and precipitation globally.

On the other hand, climate change–related factors such as sea surface temperature, ocean acidification, and sea level rise could directly affect seagrass growth and survival. The results of the mixed effects model in our results indicated that seagrasses exhibited higher seagrass EHI to increase in temperature, and were more influenced by land and climate pressures due to their sensitivity to environmental factors such as temperature, light availability, and water quality. Turschwell et al.^11^ in a review also found that seagrasses were highly dependent on adequate light penetration for photosynthesis, and changes in water clarity due to factors like sedimentation and nutrient loading can impede their productivity. Overall, the differential impacts on mangroves and seagrass by land and marine pressures versus land and climate pressures reflect their unique ecological characteristics and vulnerabilities to specific environmental stressors. This observation aligned with our result as well that the hypervolumes occupied by mangroves and seagrasses only overlap by 7%, indicating a relatively low degree of similarity or shared characteristics between these two ecotones.

Salt marshes, situated at the interface of land and sea, exhibited higher salt marsh EHI to higher sea surface temperature, and showed strong resistance to various pressures including climate, land, and marine pressures (Figure 2). This can be explained by salt marshes that have the ability to withstand tidal fluctuations and high salinity, enabling them to thrive under elevated sea levels.^10^^,^^45^ This resilience indicates that salt marshes have specific characteristics and mechanisms that allow them to recover from tidal and salinity fluctuations. Therefore, these unique adaptations may contribute to the restoration of coastal ecotones.

### Linking mangrove health to soil carbon accumulation and biodiversity

By constructing the comprehensive EHI for BCEs using our BPT model, we obtained a global map illustrating the health status of BCEs (Figure 3). By comparing the results from previously published studies with the global distribution of our EHI for BCEs, we identified some interesting observations. For example, as an important ecological indicator, mangrove soil organic carbon content was shown in the Figure S10, which was positively correlated with mangrove EHI (Figure S11A). The results of linear fitting indicated that for every increase of 0.1 in mangrove EHI, soil organic carbon (SOC) increased by 68.916 Mg per hectare (slope = 689.16, R square = 0.37, *p* value < 0.001, Figure S12). Carugati et al.^46^ also found that degraded mangroves could decrease the sedimentary organic carbon. Furthermore, our analysis revealed a positive correlation between mangrove health and species diversity (slope = 70.28, R^2^ = 0.23, p ¡ 0.001, Figure S13). Indonesia, which is identified as having the highest mangrove diversity (Figure S14),^47^ also demonstrated a significant level of mangrove health in our study (Figure 3A). This finding is consistent with IUCN^33^ and Rahman et al.,^48^ who highlighted that healthy mangroves support high species diversity. Healthy mangrove ecotones tended to have higher levels of biodiversity and a higher ability to accumulate carbon compared to degraded or disturbed mangrove areas. By comparing with the results of previous studies, we could validate and verify the effectiveness and applicability of our BPT model, which further strengthened our confidence in the index and demonstrates its value and importance in assessing the health of BCEs on a global scale.

### Highlighting the structure of BCEs: The crucial variable of BCEs conservation

As the transitional zone to maintain land and ocean, BCEs play a crucial role in maintaining the balance and stability of both environments. The results of Global Sensitivity Analysis showed that the health status of BCEs was more sensitive to ecotones fitness indicators compared to pressure indicators (Figure 5). Mangrove EHI was explained 54% by species characteristics indicator-height, while only explained 13% by marine pressure. Salt marsh EHI was explained 92% by habitat structure indicator-area, while only explained 5% by climate pressure. Seagrass EHI was explained 95% by overlap, while explained 58% and 26% by area and land pressures, respectively. Our results indicate that ecosystem structure is more critical for maintaining health status than external pressures. This result aligns with the conclusions from the mangrove risk assessment,^33^ which emphasizes maintaining ecosystem integrity is crucial for coping with the impacts of climate change. Additionally, it supports findings from Kentula et al.^31^ and Istvan et al.,^30^ which highlights the importance of preserving connectivity between marshes and adjacent ecosystems to support biodiversity. To effectively support the health and resilience of these ecotones, it is essential to prioritize efforts aimed at expanding their extent and enhancing their structures.

Compared to the structural characteristics of ecosystems, our study found that climate change and anthropogenic pressures serve as secondary influences on the health of BCEs. This suggests that robust ecosystem structures significantly enhance the resilience and recovery capacity of BCEs, allowing them to maintain better health status in the face of external pressures. This finding aligns with other studies that emphasize the critical role of habitat integrity in ecosystem resilience. For instance, research has shown that well-connected habitats can buffer against the impacts of climate change and human activities, ultimately supporting greater biodiversity and ecosystem functionality.^49^ Thus, prioritizing the preservation and enhancement of ecosystem structure is essential for effective BCE conservation and restoration efforts. However, this does not diminish the importance of pressures from land use and climate change, as previous research indicates that these factors can also affect ecosystem integrity. Consequently, while structural characteristics provide resilience, external pressures may still indirectly influence the health status of BCEs by impacting their overall integrity and connectivity.^50^ This finding also provides theoretical support and research directions for future ecosystem restoration and nature-based solutions.

### Conclusion

We developed a Benefit-Pressure-Transformation Risk model (BPT model) to assess the health status of BCEs globally or locally at any scale, which accounts for a framework for the conservation and restoration of BCEs. BCEs play a crucial connecting role, serving as vital transition zones between land and ocean. In this research, we calculated the EHI for 3 core BCEs (mangroves, salt marshes, and seagrasses) at 1°(lat) × 1°(long) (100km × 100 km at the equator) pixel scale globally. Globally, the mean EHI for mangroves is 0.55 (ranging from 0.19 to 0.83), for saltmarshes 0.51 (ranging from 0.06 to 0.94), and for seagrasses 0.50 (ranging from 0.05 to 0.86). Around 20% of BCEs fall into the low health category, highlighting an urgent need for protection. We observe that with each 1-degree increase in latitude, the EHI values for mangroves, salt marshes, and seagrasses decline by 0.007, 0.003, and 0.004, respectively. Furthermore, our analysis shows that healthier mangroves tend to have significantly higher carbon sequestration capacities and biodiversity compared to degraded or disturbed areas with low EHI. Specifically, for every 0.1 increase in EHI, SOC content rises by 68 Mg per hectare.

By identifying regional patterns of hypervolume overlap, our study offers valuable insights for prioritizing conservation efforts tailored to specific geographical contexts. We found that habitat structure, such as area and integrity, plays a more critical role in determining BCE health than environmental pressures. This finding not only underscores the importance of habitat structure in ecosystem health but also provides theoretical support and research directions for future ecosystem restoration and nature-based solutions. As global warming is expected to lead to increases in temperature and precipitation, mangroves may become more likely to expand into higher latitudes. However, it is important to note that precipitation in temperate regions may include both rainfall and snow, which could limit the extent of mangrove spread. Overall, this research provides a robust and comprehensive method for assessing the health of BCEs, offering a valuable scientific basis for their protection and restoration efforts.

### Limitations of the study

The complexity of BCEs, combined with the current limitations of global monitoring networks, necessitated the adoption of a coarse resolution for this initial global assessment. However, this coarse resolution may not capture very localized variations and specific pressures on BCEs. As such, while this study provides a critical first step for the global BCE health evaluation, future assessments could benefit from high resolution data to support more localized conservation efforts.

## Resource availability

### Lead contact

Requests for further information and resources should be directed to and will be fulfilled by the lead contact, Matteo Convertino (matteo@sz.tsinghua.edu.cn; matconv.uni@gmail.com).

### Materials availability

This study did not generate new materials.

### Data and code availability

• All the data reported in this paper will be shared by the lead contact upon request.
• This paper does not report original codes.
• Any additional information required to reanalyze the data reported in this paper is available from the lead contact upon request.

## Acknowledgments

This work was funded by [Shenzhen Pengcheng Peacock Pengcheng Talents Funding] via grant [020210320], [Shenzhen Stability Support Grant] via grant [WDZC20231128160214001], [Shenzhen Science and Technology Program For The Key Laboratory of Ecological Remediation and Carbon Sequestration at 10.13039/100018913Tsinghua Shenzhen International Graduate School] via grant [ZDSYS20220606100806014], and [Cross-disciplinary Program at Tsinghua Shenzhen International Graduate School] via grant [JC2024011]. The authors thank all members of the TREES lab for their feedback during the execution of the work.

## Author contributions

Conceptualization, M.C.; methodology, M.C., and J.Z.; investigation, M.C. and J.Z.; writing-original draft, J.Z.; writing-review and editing, M.C. and J.Z.; funding acquisition, M.C.; resources, M.C.; supervision, M.C.

## Declaration of interests

The authors declare no competing interests.

## STAR★Methods

### Key resources table

**Table:** 

REAGENT or RESOURCE	SOURCE	IDENTIFIER
**Software and algorithms**		

Python version 3.9	Python Software Foundation	https://www.python.org
R studio	R Project for Statistical Computing	https://www.r-project.org
ArcGIS10.8	ArcGIS Software	https://www.arcgis.com/index.html
Sensobol R package	R studio	Puy et al. 2022^51^
lme4 R package	R studio	Bates et al. 2015^52^
Hypervolume R package	R studio	Blonder et al.^53^

### Method details

#### Benefit-Pressure-Transformation risk model

We developed a Benefit-Pressure-Transformation Risk model (BPT model) to assess the health status of BCEs (Figure 1). The model consists of two parts: one part is to assess the benefit of BCEs (blue part of the framework) and the other is to assess the risk of BCEs (red part of the framework). We considered the benefits part in terms of ecotone fitness, which contains habitat structures, species characteristics, and ecological services. In the risk assessment, we incorporated the concept that risk encompasses hazard, vulnerability, and exposure. We defined pressures in terms of both natural and human-induced pressures as hazards and included the vulnerability of species in ecotones. The risk of transformation indicated exposure risk, which was represented by the overlap of hypervolumes. These constructed variables were combined through the BPT model to produce EHI for 2845 pixels worldwide (1°(lat) × °(long), 100km × 100 km at the equator) (Figure 1), with the higher EHI score reflecting the higher health state of ecotone (Figure 3). The calculation was conducted in Excel2022, and the map visualization was conducted in ArcGIS10.8.

##### Ecotone fitness

We measured the systemic ecotone fitness as a multicriteria function considering three features: habitat structures (area and fragment area), species characteristics (age and height) and ecological services (Aboveground Biomass (AGB) and Soil Organic Carbon (SOC)). For mangroves, the ecotone fitness was calculated by the mean value of 6 normalized parameters: area, fragment area, age, height, AGB, and SOC. For salt marsh and seagrass, ecotone fitness was determined using normalized area data due to data limitations. Our model is flexible, allowing users to choose the ecotone fitness indicators most relevant to their specific assessment needs. The ecotone fitness typically reflects the Benefits (B) it provides. Specifically, for pixel i:

(Equation 1) $$B_{i}=\sum_{n=1}^{6}\frac{Area_{i}+Fragment\;area_{i}+Age_{i}+Height_{i}+AGB_{i}+SOC_{i}}{n}$$

##### Environmental pressures

We quantified the systemic pressure from both nature and human activities, as well as the vulnerability of species within each ecotone at the pixel scale. For mangrove and seagrass, the Pressure (P) was calculated by the mean value of climate pressure (Pclimate), land pressure (Pland), marine pressure(Pmarine) and species pressure (Pspecies). For salt marsh, species pressure was not included because of lots of missing data. All the pressure parameters (climate pressure, land pressure, marine pressure, and species pressure) were all compound variables and the calculated processes were showed in Table 2. Specifically, for pixel i:

(Equation 2) $$P_{i}=\sum_{n=1}^{4}\frac{Pclimate_{i}+Pland_{i}+Pmarine_{i}+Pspecies_{i}}{n}$$

##### Risk of transformation

Hutchinson introduced the concept of the multidimensional hypervolume in 1957 to quantify species’ ecological niche space.^35^ This concept has since been applied in the quantification of ecological niches. BCEs (mangrove, salt marsh, and seagrass) were closely related to each other in spatial distribution. In this research, to investigate the overlap of BCE niche space, we analyzed the five-dimensional climatic hypervolumes (Photosynthetic Active Radiation (PAR), tidal amplitude, Sea Surface Temperature (SST), air temperature and precipitation) of three kinds of BCEs at global (Figure 6) and continental scales (Figures S4–S9; Table 3). At the continental scale, we applied a uniform transformation risk standard, and this allowed us to calculate the EHI for BCEs on a pixel scale across the globe. We hold the view, along with Hutchinson’s theory,^35^ that the higher the overlap of the hypervolume the higher the Risk of Transformation (T) among three kinds of BCEs. This is because the chance of one habitat shifting into another one is higher for purely geometrical reasons of overlap and similarity of bioclimatic features. ”Hypervolume” R package was used to calculate the hypervolume and the overlap. The calculation was conducted in R Studio 2023. For detailed information regarding the specific calculation method and application of the model, please refer to.^53^

##### Ecosystem health index (EHI)

In ecological modeling, addition is often used to represent cumulative effects, while multiplication is often used to express the effects of interactions. Therefore, the addition of cumulative effects is used to represent the fitness and pressure of the ecotone in our BPT model. Pressure and transformation risk are multiplied together, as well as the benefits (ecotone fitness) to represent the multiplicative nature of ecological processes leading to cascading ecosystem collapse. EHI of three kinds of BCEs for each pixel was calculated using our EHI model (Figure 1). The EHI for pixel i was given by:

(Equation 3) $$EHI_{i}=B_{i}\times \left[1-\left(P_{i}\times T_{i}\right)\right]$$

##### EHI classes

The EHI for three BCEs was divided into three levels using the natural breakpoint method and the specific breakpoints are shown in Table 1. EHI was then divided into three classes, specifically.

• High EHI: signifies a healthy ecotone with good ecological structure and function. The ecotone exhibits a state of balance and resilience and is capable of withstanding relatively high pressures from both natural forces and human activities. This healthy state contributes to the overall well-being of the ecotone itself and the humans who depend on its services.
• Medium EHI: signifies a relatively healthy ecotone with a moderate level of ecological structure and function. It has a degree of resilience to pressures and is able to maintain basic ecological processes and support biodiversity in the face of slight pressures from both natural forces and human activities. This health status is capturing the systemic ecological condition of ecotones and the services they provide. An ecotone in medium EHI status is possibly prone to degradation when faced with significant pressures or alteration of its structure.
• Low EHI: signifies an unhealthy ecotone with significant degradation and impairment of ecological structure and function. The ecotone may experience severe disruptions in essential processes, leading to a loss of biodiversity and services. It is highly vulnerable to environmental pressures and human activities, resulting in a limited capacity to recover or adapt. This state of low ecotone health poses challenges to sustainability and the overall well-being of the ecotone itself and the humans who depend on its services.

#### Data sources and uncertainty

The data for mangroves were obtained from the Global Mangrove Watch (GMW),^8^ which provides maps based on remote sensing and field surveys. For the calculation, the coastal wetland site polygons were intersected with the GMW mangrove extent files and overlapping polygons were dissolved to calculate the area (ha) within each coastal wetland pixel. Salt marsh data were sourced from the World Conservation Monitoring Center (WCMC), utilizing historical data and ecological assessments.^54^ The coastal wetland pixels were intersected with the WCMC salt marsh extent (polygon vector only) files and overlapping polygons were dissolved to determine the area for each site. Seagrass distribution was derived from WCMC seagrass extent (merged point and polygon vectors),^55^ where the coastal wetland pixels intersected with these extent files. Overlapping polygons were dissolved, and the area was calculated and added to the dataset. For more information on data sources of BCEs extent, pressures, and Environmental covariates and calculation processes relevant to this study, please refer to the supplementary data in Sievers et al.,^34^

While mangrove areas have been mapped extensively and with relative accuracy, the mapping and modeling of salt marsh and seagrass areas still carry significant uncertainty due to methodological challenges. The data used in these analyses can influence the assessment results obtained. In the final assessment results, the missing values were 16% for mangroves, 15% for salt marshes, and 29% for seagrasses, attributed to incomplete datasets from various sources during our EHI calculation process (Table 1). These uncertainties may impact the results of the following analysis. To address these, we have added a comparative discussion of our findings with previous studies in the discussion section to ensure the credibility of our results.

### Quantification and statistical analysis

#### Global Sensitivity Analysis

In our BPT model, numerous interacting parameters contribute to its output EHI, each with its own uncertain degree of influence. Variance-based sensitivity analysis was conducted to analyze how the input parameters of the BPT model could explain the variance of EHI by calculating the first-order Sobol indices. The “Sensitivity” R package was used to calculate the GSA. The calculation was conducted in R Studio 2023. The first-order Sobol index for each variable $X_{i}$ was calculated as:

(Equation 4) $$S_{X_{i}}=\frac{V_{i}\left[E\left(EHI|X_{i}\right)\right]}{V\left(EHI\right)}$$

where, $S_{X_{i}}$ is the first-order sobol index for i-th input parameter; $V_{i}$ [E(EHI—$X_{i}$)] represents the variance of the conditional expectation of EHI given $X_{i}$; V(EHI) is the total variance of EHI.

#### Mixed effects model for ecosystem health

Climatic factors were used to get the hypervolume overlap among BCEs and not direct input parameters in our BPT model. Therefore, to disentangle the effects of direct climate forcing on the EHI of BCEs globally, A mixed effects model was carried out to get $EHI_{c}$ as the worldwide average and not at the pixel scale like EHI. Given $EHI_{c}$ is purely a ecoclimatic baseline and services and pressures must be accounted for in a comprehensive ecological valuation, we set temperature, sea surface temperature, precipitation, PAR, tidal amplitude, and pressures from both nature and human activities as fixed effects. Considering different continents may have different patterns between $EHI_{c}$ and climatic factors, we specified random effect by different hypervolume overlap of BCEs in each continent. The ”lme4″ R package was used to calculate the mixed effects model; the calculation was conducted in R Studio 2023. The mixed effects coefficient β for variable *X*, was calculated as:

(Equation 5) $$EHI_{c}=\beta X+b+\epsilon$$

where $EHI_{c}$ is the bioclimatic predictor (considering the mixed effects model) of EHI representing the influence of climate and overlap features only. β is a row vector of coefficients for each fixed predictor, *X* is a row vector of each fixed predictor variable, *b* is a row vector of random effect intercept, ϵ is the random noise accounting for unexplained variability in the model.
