# Supplementary material for: Blueprinting the ecosystem health index for blue carbon ecotones
Source: iScience. 2024 Nov 26;27(12):111426. doi: 10.1016/j.isci.2024.111426 (PMC11664127; doi:10.1016/j.isci.2024.111426)
Supplement: Document S1. Figures S1–S14 [file mmc1.pdf]

iScience, Volume *27*

Supplemental information

Blueprinting the ecosystem health index for blue carbon ecotones

Jiaqi Zhang and Matteo Convertino

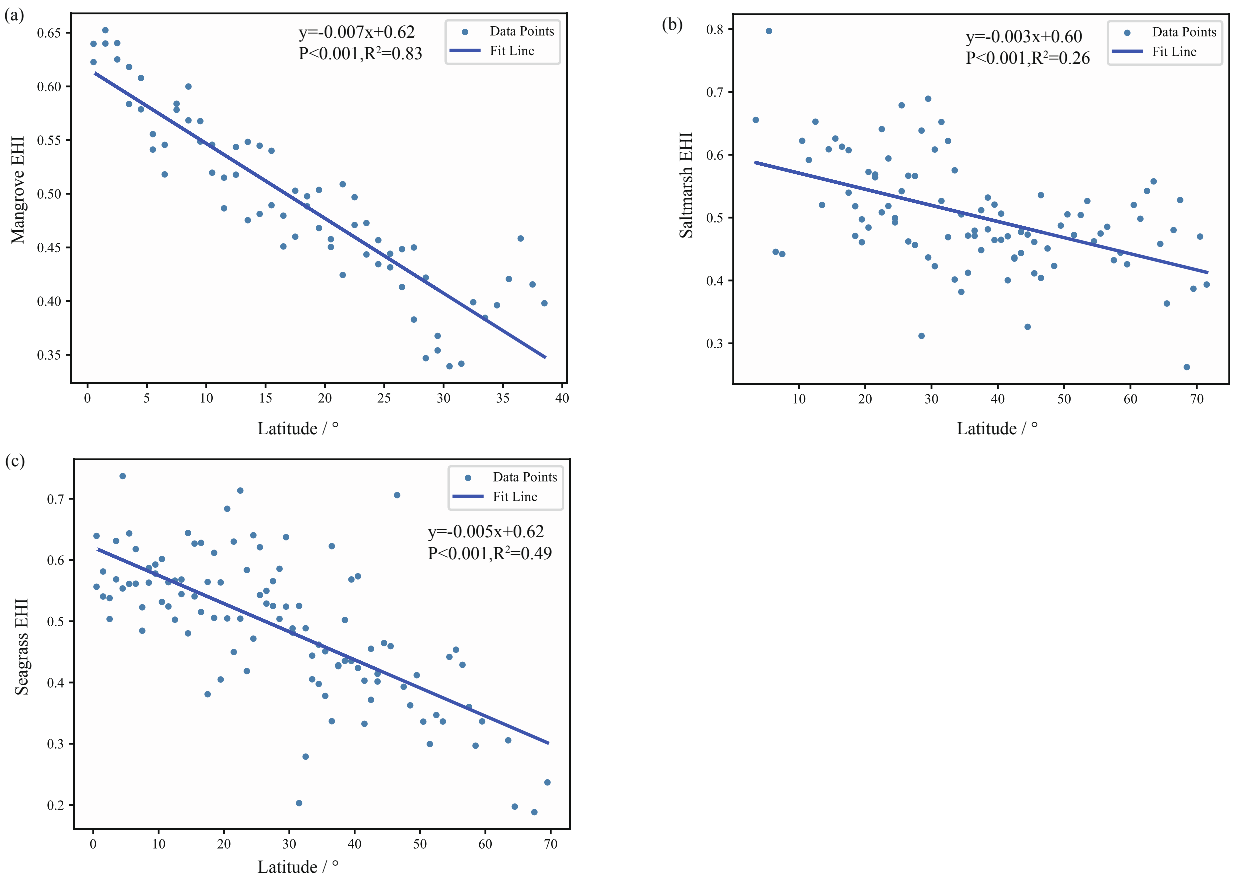

Figure S1: Latitude-Dependent Trends in Blue Carbon Ecotone EHI.
(a) Mangrove; (b) Salt marsh; (c) Seagrass.

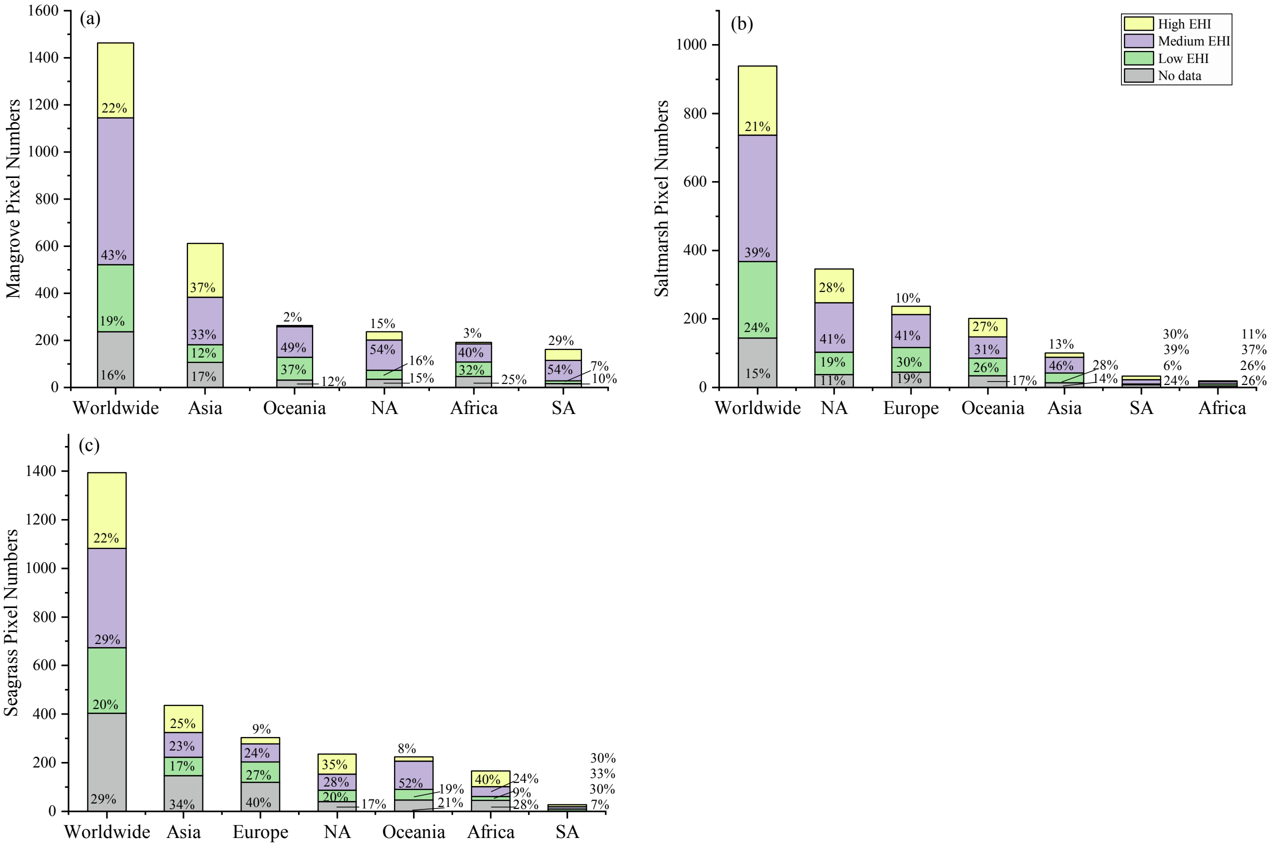

Figure S2: Three Levels of Blue Carbon Ecotones EHI.
BCE is categorized into three classes (High/Medium/Low) at global and continental scales by the natural breakpoint method. (a) Mangrove; (b) Salt Marsh; (c) Seagrass.

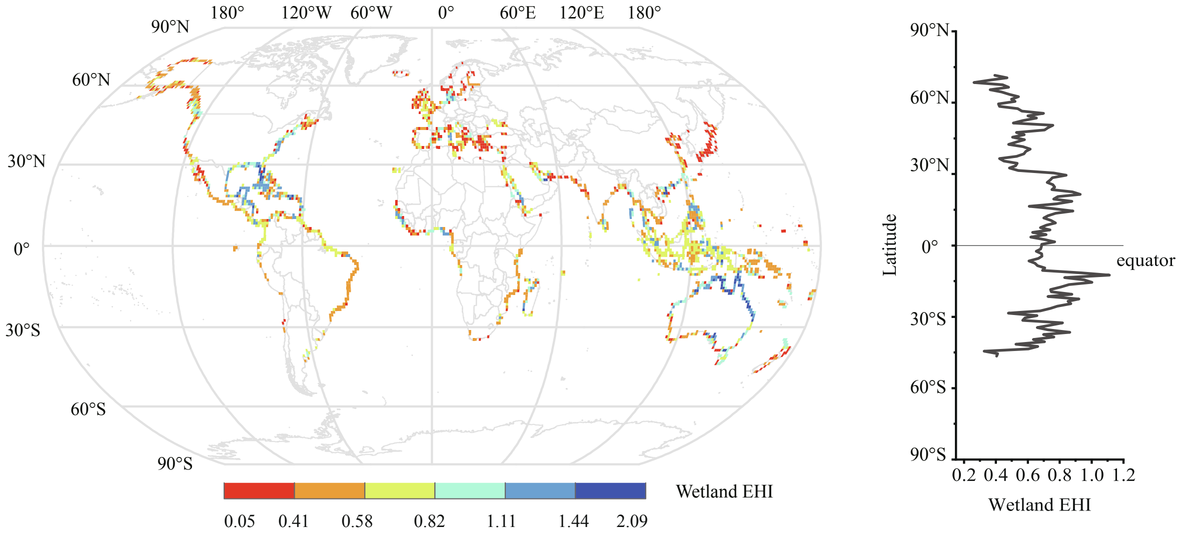

Figure S3: Global map and latitudinal pattern of coastal wetland EHI
. EHI is calculated as the sum of Mangroves, Salt marshes, and Seagrasses EHI.

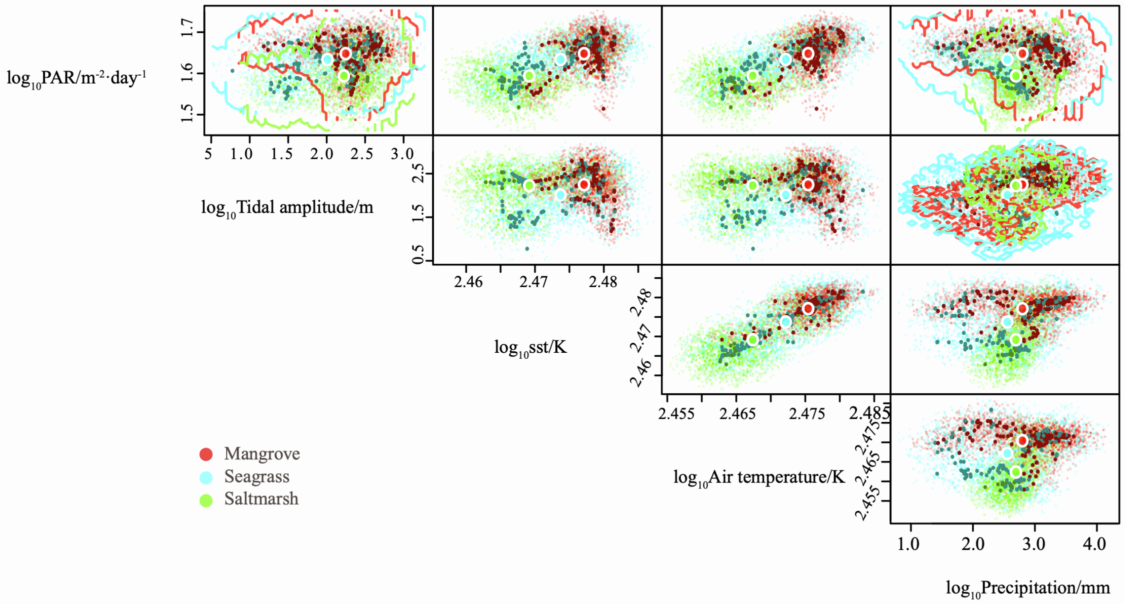

Figure S4: Pair plots for estimated climate hypervolumes of BCEs in Africa
Red: Mangrove; Blue: Seagrass; Green: Salt Marsh. Hypervolumes were constructed using a Gaussian kernel density estimate with the Silverman method. All variables have been log10-transformed. The colored points for each ecotone reflect the stochastic description of each hypervolume.

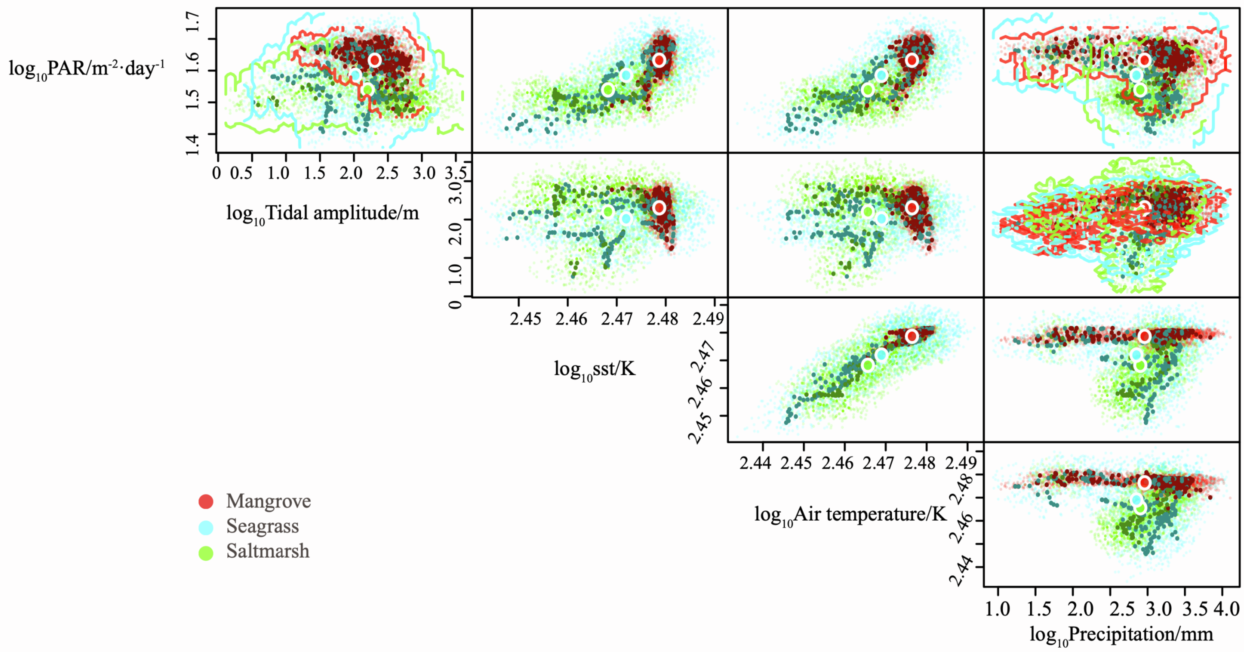

Figure S5: Pair plots for estimated climate hypervolumes of BCEs in Asia. Red: Mangrove; Blue: Seagrass; Green: Salt Marsh. Hypervolumes were constructed using a Gaussian kernel density estimate with the Silverman method. All variables have been log10-transformed. The colored points for each ecotone reflect the stochastic description of each hypervolume.

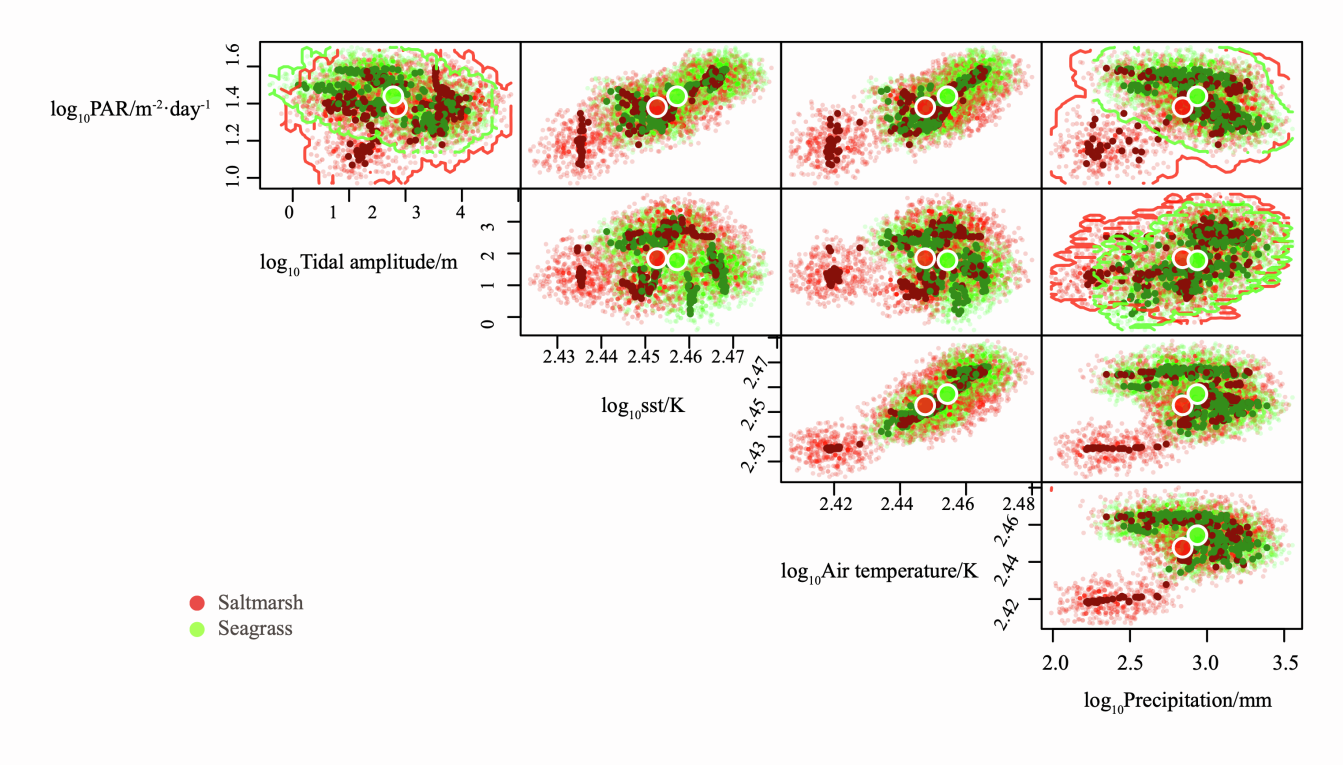

Figure S6: Pair plots for estimated climate hypervolumes of BCEs in Europe.
Red: Salt Marsh; Green: Seagrass. Hypervolumes were constructed using a Gaussian kernel density estimate with the Silverman method. All variables have been log10-transformed. The colored points for each ecotone reflect the stochastic description of each hypervolume.

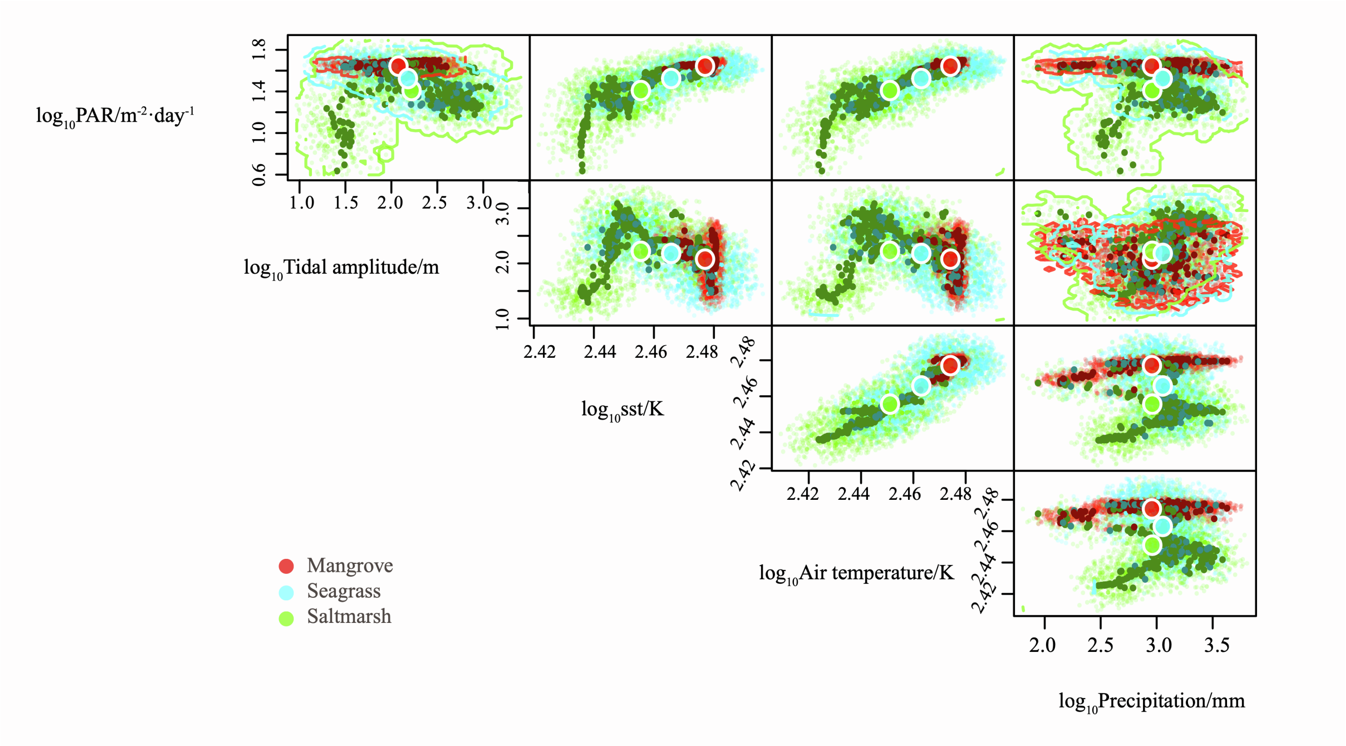

Figure S7: Pair plots for estimated climate hypervolumes of BCEs in North America. Red: Mangrove; Blue: Seagrass; Green: Salt Marsh. Hypervolumes were constructed using a Gaussian kernel density estimate with the Silverman method. All variables have been log10-transformed. The colored points for each ecotone reflect the stochastic description of each hypervolume.

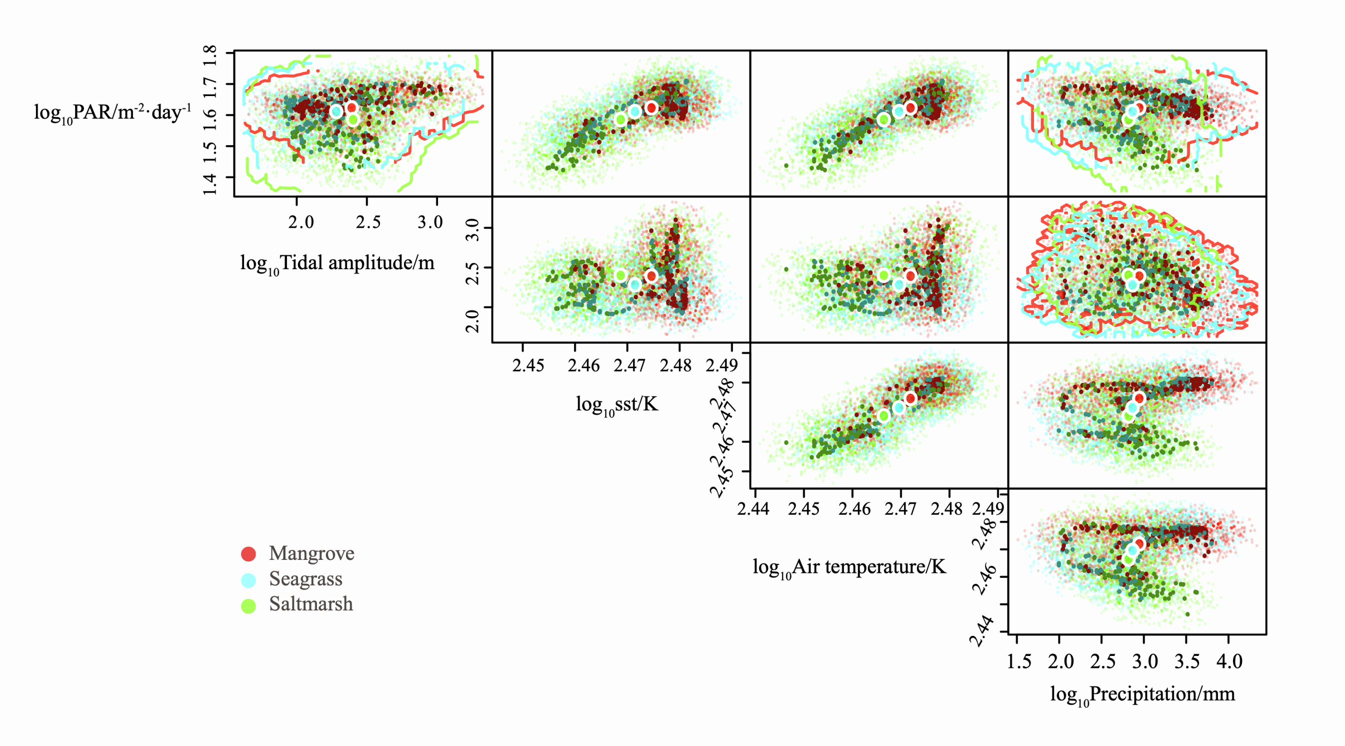

Figure S8: Pair plots for estimated climate hypervolumes of BCEs in Oceania. Red: Mangrove; Blue: Seagrass; Green: Salt Marsh. Hypervolumes were constructed using a Gaussian kernel density estimate with the Silverman method. All variables have been log10-transformed. The colored points for each ecotone reflect the stochastic description of each hypervolume.

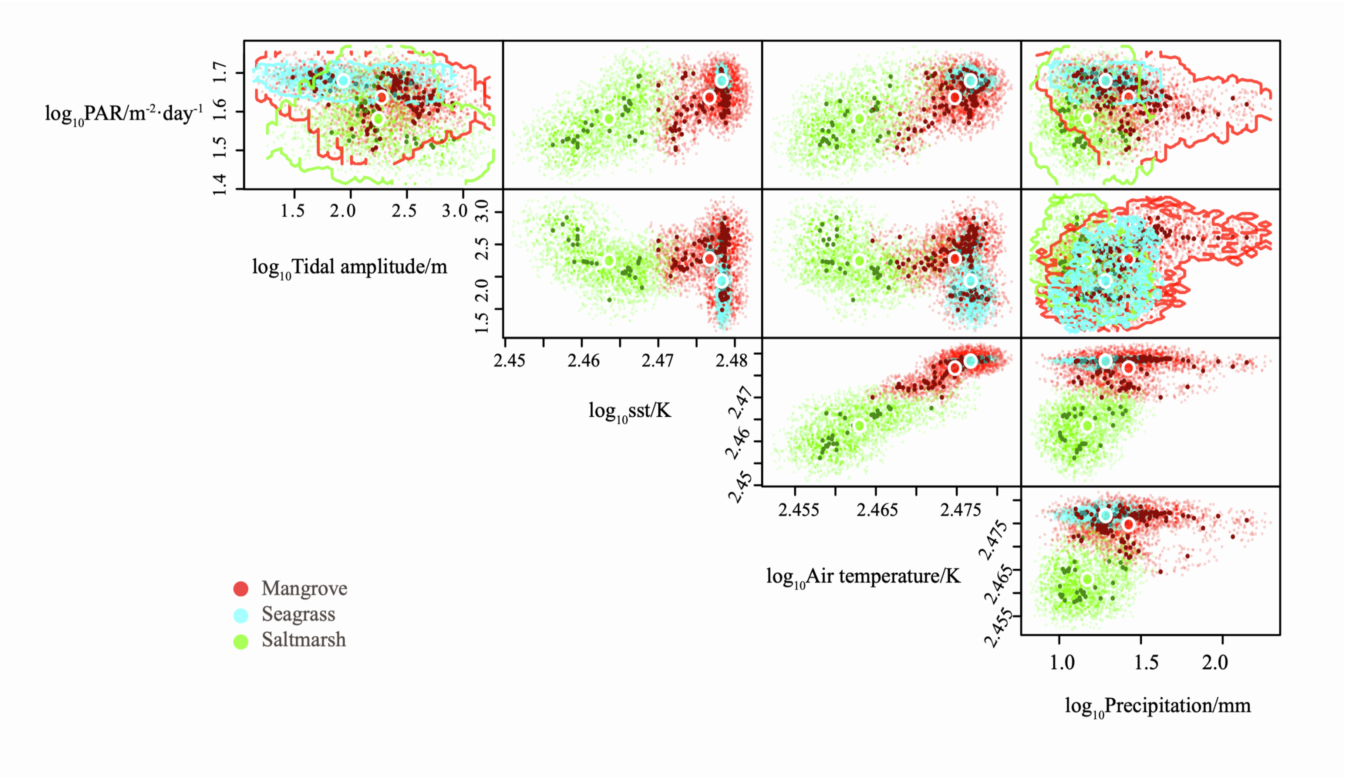

Figure S9: Pair plots for estimated climate hypervolumes of BCEs in South America. Red: Mangrove; Blue: Seagrass; Green: Salt Marsh. Hypervolumes were constructed using a Gaussian kernel density estimate with the Silverman method. All variables have been log10-transformed. The colored points for each ecotone reflect the stochastic description of each hypervolume.

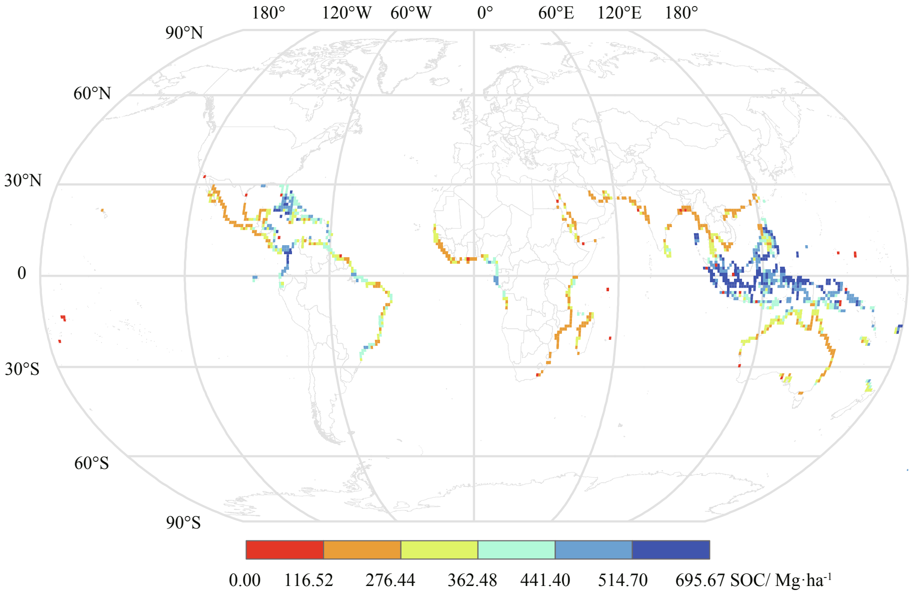

Figure S10: Global map of mangrove soil organic carbon content.

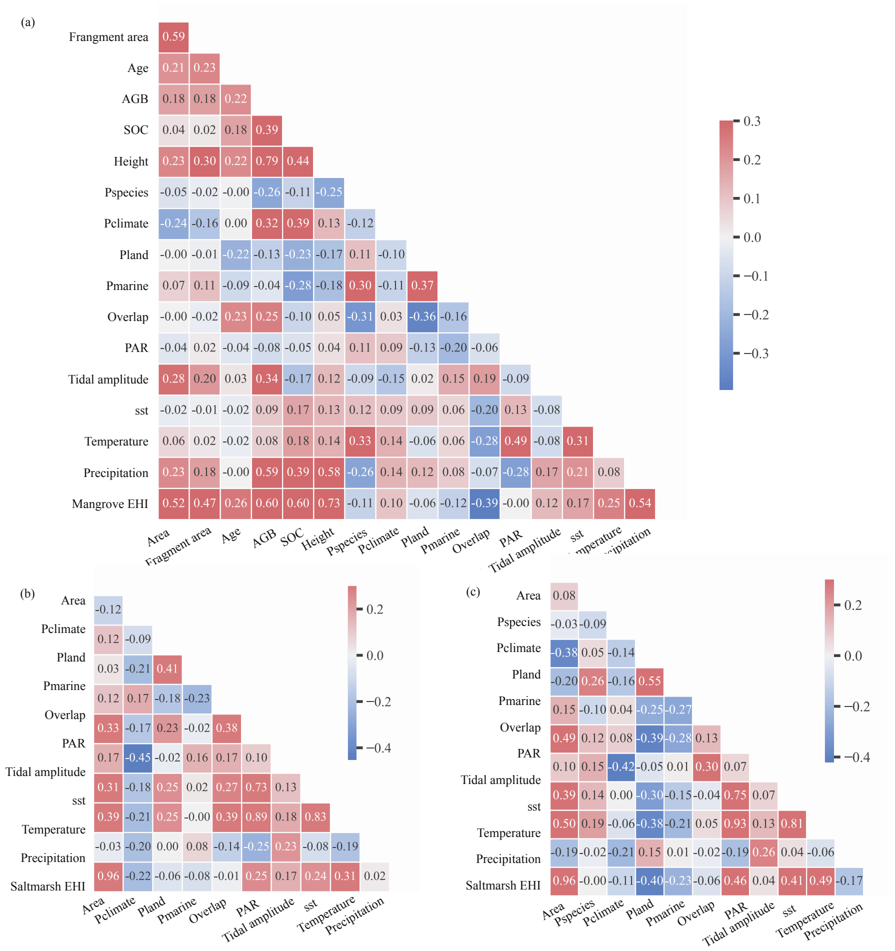

Figure S11: Pearson correlation coefficient between EHI and its determinants. Red represents positive relationships and blue negative, with the color intensity indicating relationship strength. AGB: Aboveground Biomass; SOC: Soil Organic Carbon; Pspecies: Species Pressure; Pland: Land Pressure; Pclimate: Climate Pressure; Pmarine: Marine Pressure; PAR: Photosynthetic Active Radiation; SST: Sea Surface Temperature. (a) Mangrove; (b) Salt Marsh; (c) Seagrass.

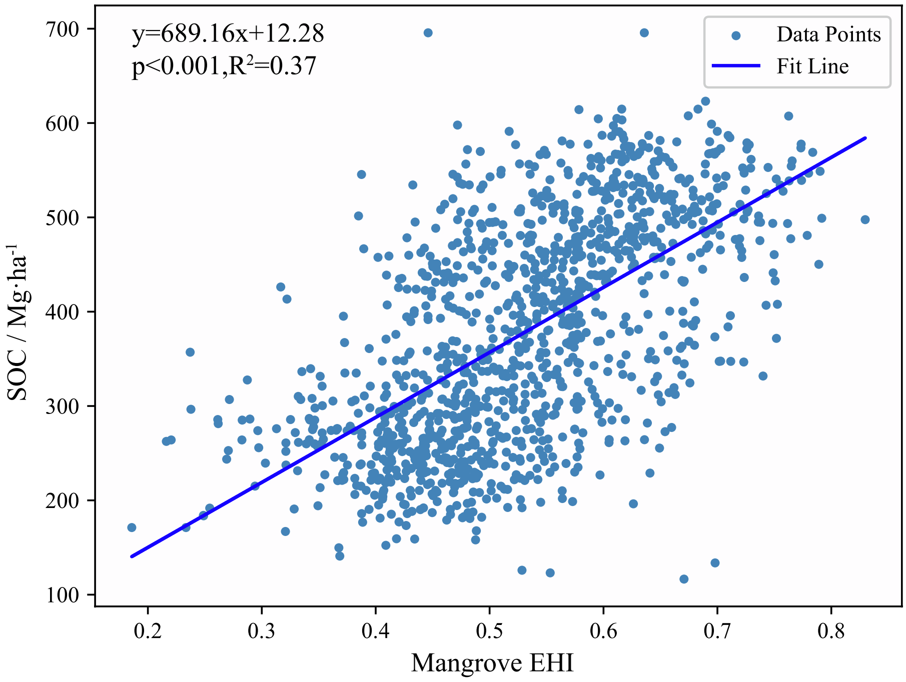

Figure S12: Relationship between Mangrove EHI and soil organic carbon.

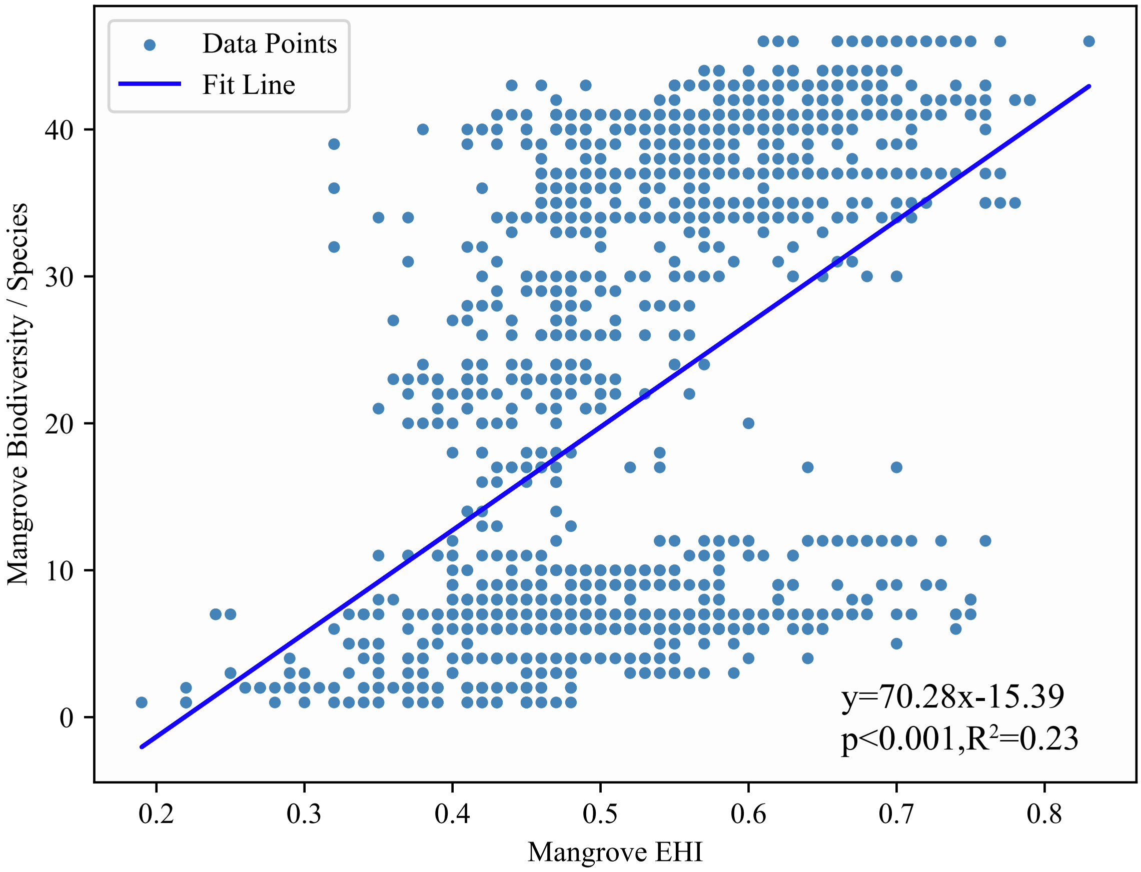

Figure S13: Relationship between Mangrove EHI and mangrove biodiversity.

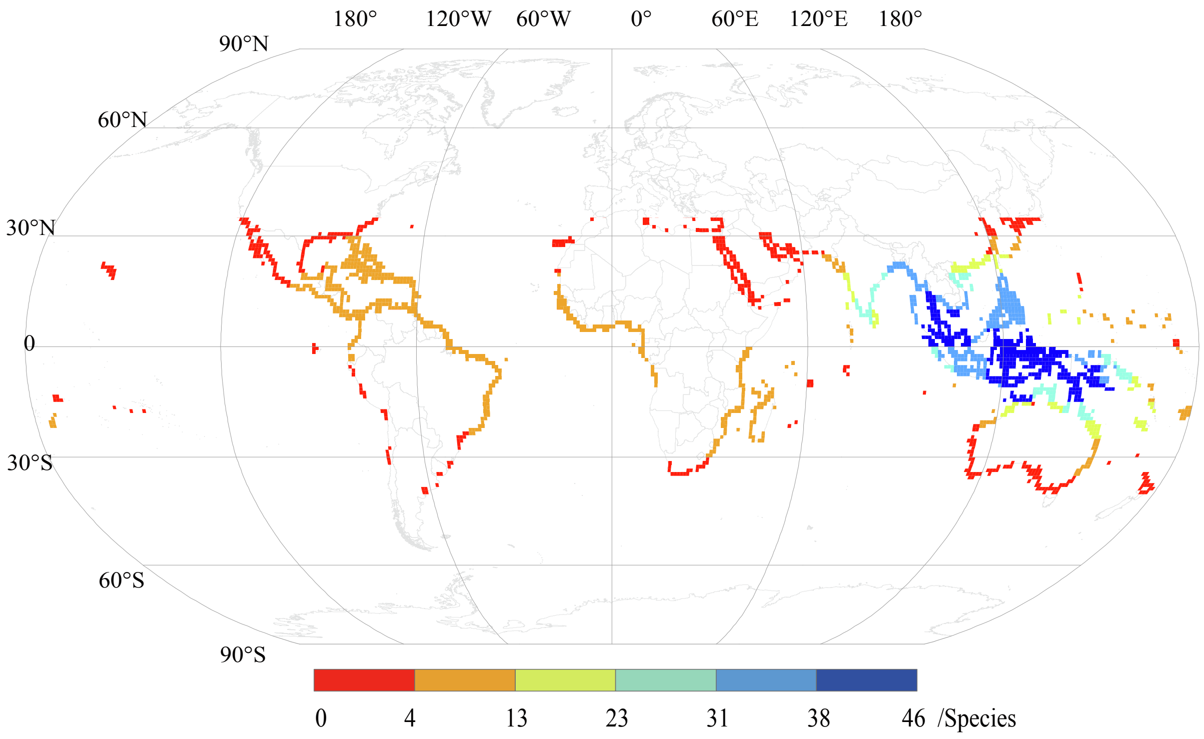

Figure S14: Global map of mangrove biodiversity.
Data were from Hamilton et al.[47]
